# The benefit of tumor molecular profiling on predicting treatments for colorectal adenocarcinomas

**DOI:** 10.18632/oncotarget.24257

**Published:** 2018-01-16

**Authors:** Philip Carter, Costi Alifrangis, Pramodh Chandrasinghe, Biancastella Cereser, Lisa Del Bel Belluz, Cosimo Alex Leo, Nina Moderau, Neha Tabassum, Janindra Warusavitarne, Jonathan Krell, Justin Stebbing

**Affiliations:** ^1^ Department of Surgery and Cancer, Imperial College, London, UK; ^2^ Department of Medical Oncology, Imperial College, London, UK; ^3^ Department of Surgery, University of Kelaniya, Kelaniya, Sri Lanka; ^4^ Department of Colorectal Surgery, St Mark’s Hospital, London, UK

**Keywords:** tumor profiling, colorectal adenocarcinoma, cancer treatment

## Abstract

We evaluated the benefit of tailoring treatments for a colorectal adenocarcinoma cancer cohort according to tumor molecular profiles, by analyzing data collected on patient responses to treatments that were guided by a tumor profiling technology from Caris Life Sciences. DNA sequencing and immunohistochemistry were the main tests that predictions were based upon, but also fragment analysis, and *in situ* hybridization. The status of the IHC biomarker for the thymidylate synthase receptor was a good indicator for future survival. Data collected for the clinical treatments of 95 colorectal adenocarcinoma patients was retrospectively divided into two groups: the first group was given drugs that always matched recommended treatments as suggested by the tumor molecular profiling service; the second group received at least one drug after profiling that was predicted to lack benefit. In the matched treatment group, 19% of patients were deceased at the end of monitoring compared to 49% in the unmatched group, indicating a benefit in mortality by tumor molecular profiling colorectal adenocarcinoma patients.

## INTRODUCTION

Colorectal cancer (CRC) is the third most prevalent cancer globally. Over a million cases are diagnosed each year and there are almost 700,000 deaths due to it annually [1–3]. It occurs more in developed countries [4, 5], with the highest rates in Australia, New Zealand, Europe and the USA.

CRC risk is strongly related to age and sex, and is most common in men and older people. Only a small proportion of colorectal cancers are thought to be due to inherited genetic abnormalities, while lifestyle factors such as diet, obesity, smoking, and lack of physical activity are much more influential. In about 20% of all cases there is a family history of colon cancer, and around 4% are due to the inherited genetic disorders familial adenomatous polyposis (FAP) and hereditary non-polyposis colon cancer (HNPCC) [4].

The acquisition of somatic mutations in oncogenes such as *KRAS*, *RAF*, and *PI3K* are often seen in colon cancer. Upregulation of the WNT and TGF-β signaling pathways results in increased activity of MYC, an important effector of colorectal cancer [6]. However, epigenetic changes are more frequent in colon cancer than mutations in genes; often a colon tumor has only one or two oncogene driver mutations, one to five tumor suppressor driver mutations, with around sixty passenger mutations, while there are hundreds of epigenetic changes. CpG island methylation of the DNA sequences encoding miR-34b/c, miR-124a, miR-137 and miR-342 resulting in their reduced expression can affect expression of hundreds of target genes in each case and are associated with colorectal cancer [7, 8, 9]. Hypermethylation or hypomethylation of CpG islands and changes in histones and chromosomal architecture are other (epigenetic) changes that can cause colorectal cancers. The development of clinical sequencing has enabled pre-treatment sequencing of relevant MAPK pathway genes such as *KRAS*, *BRAF* and *MEK1*, that predict response to EGFR targeted therapies such as cetuximab and panitumumab [10].

Guiding treatments using characterization of tumor biomarkers such as immunohistochemistry and genomic sequencing across many cancer types has resulted in better outcomes [11, 12]. We looked at the efficacy of one such method (outside the context of clinical *KRAS* and *BRAF* sequencing) from Caris Life Sciences, for colorectal adenocarcinomas. The effect of this profiling approach on overall survival and drug use was assessed.

## RESULTS

### Patient characteristics

Data describing advanced colorectal adenocarcinoma patients who underwent treatment was divided into two groups depending upon if treatments matched recommendations that used tumor molecular profiles. In the matched group, 42 patients received at least one recommended drug subsequent to collection for profiling and none that were not. In the unmatched group 53 patients all received one or more drugs predicted by profiling to lack benefit after sample collection. Patients and their tumors are summarized in Table 1.

**Table 1:** Matched and unmatched groups compared against all patients

Group	Patient & Tumor Information						
	Age	Ethnicity	Histology	Grade	Stage	Survival (days)	Mortality
All patients (95)	59.9	White: 80; Black/African American: 10;	Adenocarcinoma, NOS: 69;	Grade 2 / Moderately differentiated: 63 (66%);	IV: 38 (40%);	497	36%
		Asian: 2; Hawaiian/Pacific Islander: 1;	Mucinous adenocarcinoma: 11;	Grade 3/ Poorly differentiated: 16 (17%);	III no IIIC: 23 (24%);		
		Other/Unknown: 2	Adenocarcinoma, intestinal type: 9;	Unknown / Not Determined: 9 (10%);	IIIC: 11 (12%);		
			Adenocarcinoma in adenomatous polyp: 2;	Grade 1 / Well differentiated: 3 (3%);	II: 9 (10%);		
			Squamous cell carcinoma, NOS: 2;	Grade 4 / Undifferentiated: 2 (2%);	I: 6 (6%);		
			Signet ring cell carcinoma: 1;	None / Not Applicable: 2 (2%)	Unknown: 8 (8%)		
			Tubular adenoma, NOS: 1				
Matched only (42)	60.3	White: 32; Asian: 1; Black/African American: 8;	Adenocarcinoma, NOS: 29;	Grade 2 / Moderately differentiated: 29 (69%);	IV: 14 (33%);	442	19%
		Hawaiian/Pacific Islander: 1;	Adenocarcinoma, intestinal type: 5;	Unknown / Not Determined: 6 (14%);	III no IIIC: 13 (31%);		
		Other/Unknown: 0	Mucinous adenocarcinoma: 3;	Grade 3/ Poorly differentiated: 4 (10%);	IIIC: 3 (7%);		
			Squamous cell carcinoma, NOS: 2;	Grade 4 / Undifferentiated: 2 (5%);	II: 4 (9%);		
			Adenocarcinoma in adenomatous polyp: 2;	None / Not Applicable: 1 (2%)	I: 4 (9%);		
			Signet ring cell carcinoma: 1		Unknown: 4 (9%)		
Unmatched (53)	59.5	White: 48; Black/African American: 2;	Adenocarcinoma, NOS: 40;	Grade 2 / Moderately differentiated: 34 (64%);	IV: 24 (45%);	541	49%
		Asian: 1; Hawaiian/Pacific Islander: 0;	Mucinous adenocarcinoma: 8;	Grade 3/ Poorly differentiated: 12 (22%);	III no IIIC: 10 (19%);		
		Other/Unknown: 2	Adenocarcinoma, intestinal type: 4;	Unknown / Not Determined: 3 (6%);	IIIC: 8 (15%);		
			Tubular adenoma, NOS: 1	Grade 1 / Well differentiated: 3 (6%);	II: 5 (9%);		
				None / Not Applicable: 1 (2%)	I: 2 (4%);		
					Unknown: 4 (8%)		

### Treatment analysis

**Figure 1:**
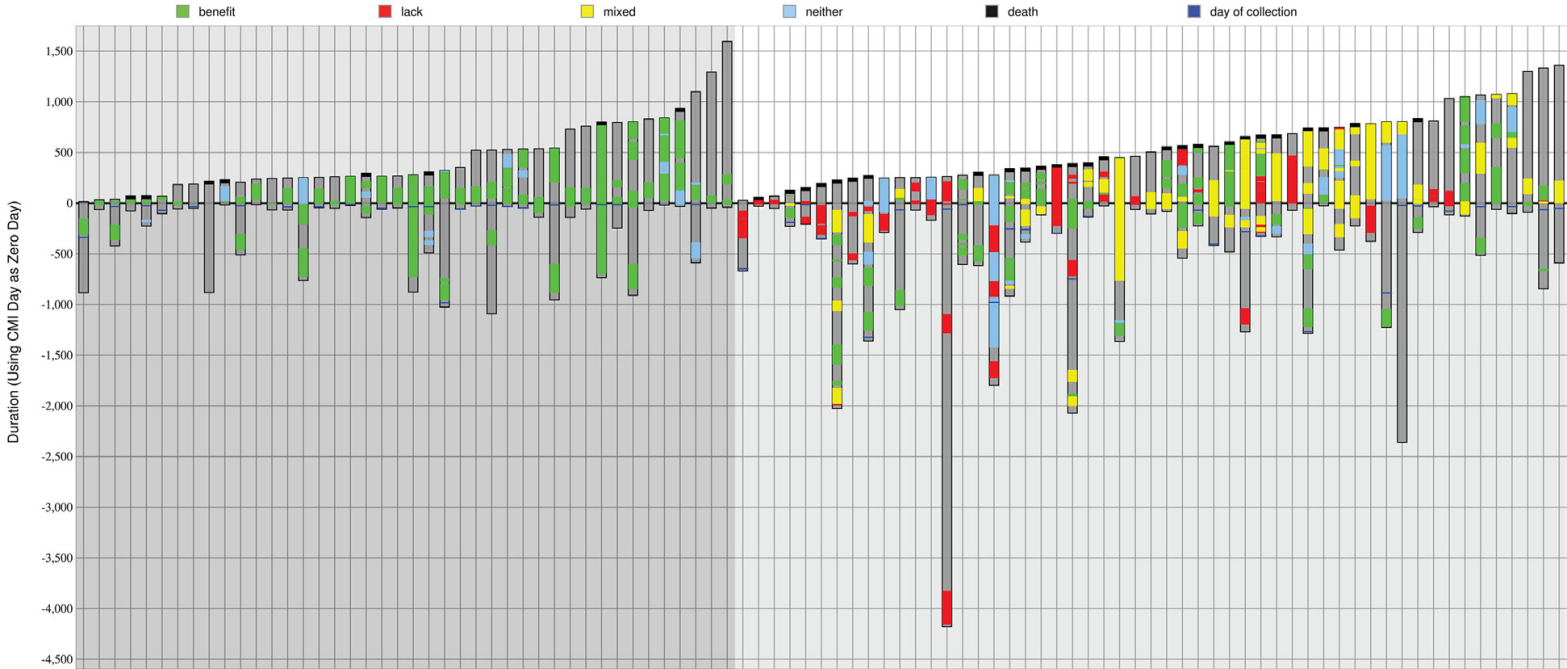
Summary of drug treatments and outcomes Treatment schedules are shown in ascending post-profiling survival time for 42 matched (on the left, darker gray background) and 53 unmatched patients (on the right, lighter gray background). A black line at the top of a bar shows that the patient was deceased. Dark gray within a bar is time monitored to either death or last follow-up. Green is time on a drug of benefit. Red is a lack of benefit treatment. Yellow is a combination therapy composed of both benefit and lack of benefit drugs. Blue is a neutral therapy (neither benefit nor lack of benefit).

Waterfall plots showing drugs received and survival for both treatment groups are shown in Figure 1. The clinical information for the 42 matched and 53 unmatched patients are depicted as columns (on the left and right of the figure respectively), where green shows administration of drugs expected to be of benefit, red is drugs that lack benefit, and yellow is both of these types in combination.

The drugs that were given are shown in Table 2. The number of patients that received a particular drug is shown in the first column, and the number of continuous treatment periods of a drug is shown in all other columns, i.e. treatments of the same patient with an intervening time are counted separately. The drugs given to the most number of patients were fluorouracil (87 patients), oxaliplatin (81), leucovorin calcium (77), bevacizumab (52) and irinotecan hydrochloride (47).

**Table 2:** Most common drugs given more than once, and those that were profiled to be of benefit, of no benefit, or classed as neither of these

Number of Patients	Most Frequently Administered Drugs (Total Treatment Periods)							
All patients treated	All patients (95) – treatment periods	Matched only patients (42), all treatments	Matched, after profiling treatments only	Unmatched patients (53), all treatments	Unmatched, after profiling treatments only	Drugs predicted of benefit	Drugs predicted to lack benefit	Drugs with no prediction (neither of benefit or lack of benefit)
fluorouracil – 87 patients	fluorouracil = 168	fluorouracil = 47	fluorouracil = 16	fluorouracil = 121	fluorouracil = 49	fluorouracil = 115	irinotecan hydrochloride = 34	leucovorin calcium = 120
oxaliplatin – 81 patients	leucovorin calcium = 127	oxaliplatin =36	bevacizumab = 13; oxaliplatin = 13	leucovorin calcium = 92	leucovorin calcium = 39	oxaliplatin = 46	oxaliplatin ***=*** 28	bevacizumab = 59
leucovorin calcium – 77 patients	oxaliplatin = 109	leucovorin calcium = 35	-	bevacizumab = 75	irinotecan hydrochloride = 27	bevacizumab = 33	fluorouracil = 25	oxaliplatin = 26
bevacizumab – 52 patients	bevacizumab = 96	bevacizumab = 21	leucovorin calcium = 12	oxaliplatin = 73	bevacizumab = 24	irinotecan hydrochloride = 21	cetuximab = 9	capecitabine = 24
irinotecan hydrochloride – 47 patients	irinotecan hydrochloride = 62	capecitabine = 18	capecitabine = 9	irinotecan hydrochloride = 49	oxaliplatin = 16	capecitabine = 14	capecitabine = 5	fluorouracil = 18
capecitabine – 31 patients	capecitabine = 47	irinotecan hydrochloride = 13	irinotecan hydrochloride = 7	capecitabine = 29	cetuximab = 13	cetuximab = 6	panitumumab = 3	ziv-aflibercept = 9
cetuximab – 15 patients	cetuximab = 17	l-leucovorin = 2; ziv-aflibercept = 2	l-leucovorin = 2; ziv-aflibercept = 2	cetuximab = 16	capecitabine = 7	doxorubicin hydrochloride = 2	-	l-leucovorin = 6
ziv-aflibercept – 8 patients	ziv-aflibercept = 9	-	-	ziv-aflibercept = 7	ziv-aflibercept = 6	-	-	cyclophosphamide = 3
l-leucovorin; panitumumab – 6 patients	l-leucovorin = 6; panitumumab = 6	-	-	panitumumab = 5	panitumumab = 3; l-leucovorin = 3	-	-	cetuximab = 2; irinotecan hydrochloride = 2; everolimus = 2; placebo = 2
-	-	-	-	l-leucovorin = 4	-	-	-	-

Patients received 6.63 treatments on average; 38% were profiled to be beneficial, 17% had no benefit, and 45% were neither of these. Matched patients had 4.17 treatments on average; 53% of these were predicted of benefit, 0% no benefit, and 47% neither. Unmatched patients had an average of 8.58 drug treatments; 33% of which were of benefit, 23% lacked benefit and 44% neither of these. 8% of unmatched patients had one or more drug treatments of benefit, and 7% had at least two of these types of treatment. Before profiling, patients received 3.92 lines of treatment on average.

The drugs that were profiled as beneficial that were most often given were fluorouracil (115 treatments), oxaliplatin (46), bevacizumab (33), irinotecan hydrochloride (21), and capecitabine (14). The drugs lacking benefit that were most commonly prescribed were irinotecan hydrochloride (34 times), oxaliplatin (28), fluorouracil (25), and cetuximab (9).

Some of the drugs did not have a recommendation, and this neither category constituted 47% of treatments in the matched group and 44% in the unmatched cohort. Of this type, leucovorin calcium was given most often - 120 times, which was 19% of all treatments.

**Figure 2:**
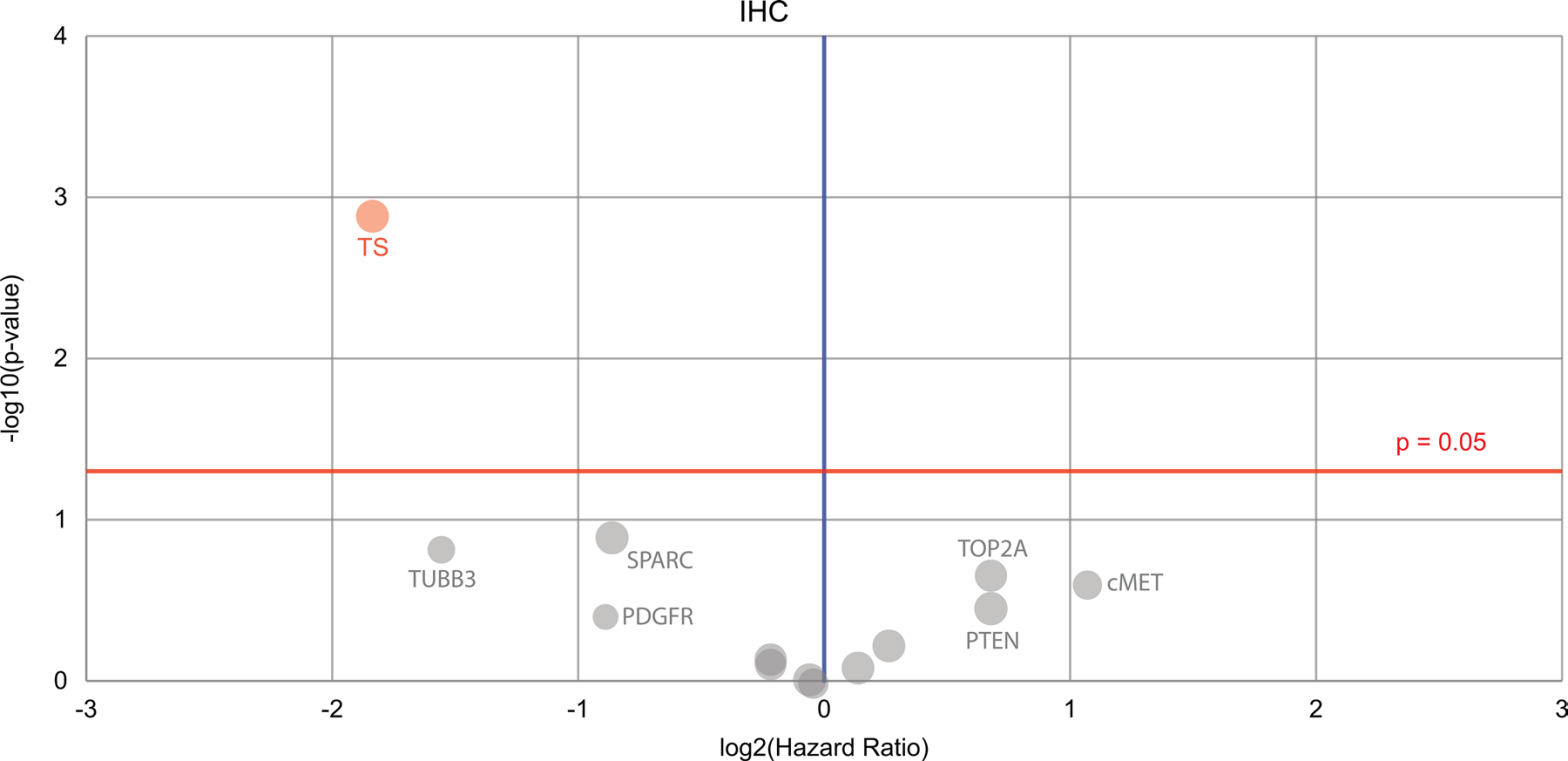
A Volcano plot is shown denoting the biomarkers’ prognostic value Only one biomarker of significance is found (on the top left), the immunohistochemistry thymidylate synthase (TS) marker. Red circle = the hazard rate of a positive biomarker result is significantly higher than that of a negative biomarker result, gray = the difference between a positive biomarker result and a negative biomarker result is not significant. Red line = significance threshold.

Fluorouracil was given far more often when it was profiled to be of benefit - 115 times that it was given coincided with beneficial predictions, whereas 25 did not. Oxaliplatin was given for 46 periods of time when predicted to be useful, whereas 28 treatments were expected to lack benefit, and 26 neither. Irinotecan hydrochloride was less optimal in its use, with 21 beneficial treatments, 34 lacking benefit, and two being of neither type. Interestingly, given the reliance on 5FU related compounds in the systemic treatment of CRC, we found that thymidylate synthase (TS) was a marker for worse outcomes in CRC (Figure 2). The literature already documents worse outcomes with high levels of TS [13]. However, only patients with unmatched treatments had positive biomarker results when IHC testing for TS.

Mismatch repair (MMR) deficient cancers are targets for anti-PD-1 therapy [14], and recently the FDA has approved Keytruda (pembrolizumab) for unresectable or metastatic solid tumors that have been identified as having a biomarker for MSI-H (microsatellite instability-high) or MMR deficient. We observe that although only three patients in this cohort had IHC markers tested for PD-L1 and the mismatch repair related markers MSH2, MSH6, PMS2 and MLH1; in all cases PD-L1 was negative while all of the MMR markers were positive.

### Survival analysis

**Figure 3:**
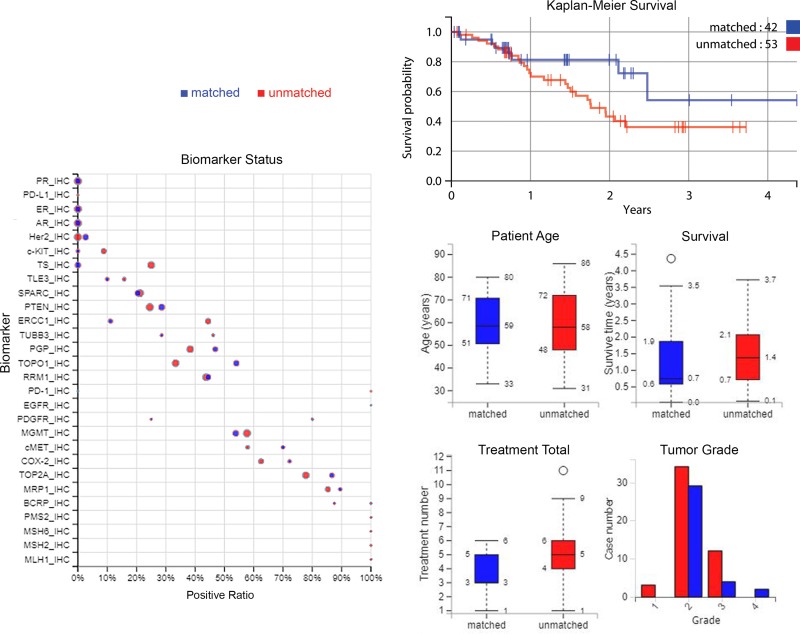
Plots of biomarker statuses, survival, and patient and tumor characteristics *Left*: biomarker statuses in the treatment groups, where the size of the circle indicates the number of cases. *Top-right*: A Kaplan–Meier curve of overall survival from time of profiling, comparing treatment groups. *Middle-right to lower-right*: a summary of patient ages, survival time, treatment numbers, and grade of samples. Blue = matched patients, red = unmatched patients.

In the matched group 19% of patients were deceased by the end of monitoring, and 49% of the unmatched group (*P* = 0.0022). The matched group survived for 442 days on average and the unmatched survived for 541 days (*P* = 0.1773) after profiling. A Kaplan–Meier curve in Figure 3 (top-right) shows the overall survival for matched patients and unmatched patients. There is also a survival plot in Figure 3 (mid-right). The matched group have a lower mortality, but survive on average for less time after profiling when measuring up to the last time of monitoring. However, it can be seen that the matched group shown on the left of Figure 1 have been monitored for less time overall; the matched group’s average number of contact days after diagnosis is 733, and the unmatched group’s is 1150 days. This may explain why in this cohort mortality is lower for matched treatments while time that patients are known to have survived is lower.

Biomarker values are compared between matched and unmatched in Figure 3 also (on the left), and some demographic and tumor information is summarized (middle-right to lower-right).

## DISCUSSION

Here we looked at the benefit of profiling tumors using biomarkers to tailor clinical therapies accordingly, by investigating the differences in response between patients that followed such recommendations to those that did not completely adhere to them. We used clinical data for a colorectal adenocarcinoma cohort of patients, whose clinicians received treatment suggestions that used tumor molecular profiling by Caris Life Sciences and received associated treatment predictions.

The unmatched group received 6.6 more drug therapies than the matched group on average, and had a poorer survival prognosis, although these patients did have tumors that were more advanced than in the matched group, as shown in Table 2. We find that thymidylate synthase (TS) as an immunohistochemical marker is associated with significantly worse outcomes in this cohort (Figure 2).

The survival curves for the different treatment groups overlap but then diverge. This indicates that therapy predictions guided by tumor profiling have a positive effect on the choice of therapies and leads to an improved outcome, as would be expected if correct stratification of treatments occurs in the clinic. A reduction in mortality was also detected. Overall this gives a good indication that there is a benefit from tumor molecular profiling in this colorectal adenocarcinoma cohort using this technology.

## MATERIALS AND METHODS

The Caris CODE database (version 1.0) contains tumor molecular profile data for 841 patients with solid tumors. It also contains demographic information about these patients, the drug treatments that they received before and after molecular profiling and records of their clinical outcomes while they were still being monitored. There are 95 colorectal adenocarcinoma patients within this database, and this colorectal cancer cohort was mined after web scraping the data from the Caris CODE website, to understand if molecular characterization affected drug selection by treating physicians, and if any molecular subsets had different outcomes across tumor types. Table 1 describes the clinical characteristics of the patients that were profiled. According to Caris Life Sciences, 36% of cases used here had a metastatic sample profiled.

As shown in Figure 1, the amount of time that patients were monitored varied, although on average patients’ treatment records were available for 966 days (733 for matched treatment patients, 1150 for unmatched patients), and on average the time of monitoring after profiling was 497 days. The longest amount of time that records were available, i.e. before and after diagnosis, up until the last contact day, was 4442 days. The longest period of monitoring after tumor profiling (the patient represented on the furthest right of Figure 1) was 1594 days; this was 1634 days after diagnosis.

## Abbreviations

CRC: colorectal cancer
FAP: familial adenomatous polyposis
HNPCC: hereditary non-polyposis colon cancer
TS: thymidylate synthase
