# Correction: The benefit of tumor molecular profiling on predicting treatments for colorectal adenocarcinomas

**DOI:** 10.18632/oncotarget.24690

**Published:** 2018-03-13

**Authors:** Philip Carter, Costi Alifrangis, Pramodh Chandrasinghe, Biancastella Cereser, Lisa Del Bel Belluz, Cosimo Alex Leo, Nina Moderau, Neha Tabassum, Janindra Warusavitarne, Jonathan Krell, Justin Stebbing

**Affiliations:** ^1^ Department of Surgery and Cancer, Imperial College, London, UK; ^2^ Department of Medical Oncology, Imperial College, London, UK; ^3^ Department of Surgery, University of Kelaniya, Kelaniya, Sri Lanka; ^4^ Department of Colorectal Surgery, St Mark’s Hospital, London, UK

**This article has been corrected:** The proper Materials and Methods and Conflicts of Interest information is as follows:

**MATERIALS AND METHODS**

The Caris CODE database (version 1.0) contains tumor molecular profile data for 841 patients with solid tumors. It also contains demographic information about these patients, the drug treatments that they received before and after molecular profiling and records of their clinical outcomes while they were still being monitored. There are 95 colorectal adenocarcinoma patients within this database, and this colorectal cancer cohort was mined after web scraping the data from the Caris CODE website, to understand if molecular characterization affected drug selection by treating physicians, and if any molecular subsets had different outcomes across tumor types. Table 1 describes the clinical characteristics of the patients that were profiled. According to Caris Life Sciences, 36% of cases used here had a metastatic sample profiled.

As shown in Figure 1, the amount of time that patients were monitored varied, although on average patients’ treatment records were available for 966 days (733 for matched treatment patients, 1150 for unmatched patients), and on average the time of monitoring after profiling was 497 days. The longest amount of time that records were available, i.e. before and after diagnosis, up until the last contact day, was 4442 days. The longest period of monitoring after tumor profiling (the patient represented on the furthest right of Figure 1) was 1594 days; this was 1634 days after diagnosis.

The data were analysed independently of Caris. Patients were covered under 1 of 4 different protocols or exemptions, listed as follows. (1). The Caris Registry Protocol (TCREG-001-00-V2-1209) was approved by WIRB (WIRB Tracking #20092285) and has an NCT# of NCT02678754. (2). The Caris POA Prospective Repository (COE-001-0815) was approved by WIRB (WIRB Tracking #20162864) and has an NCT# of NCT03324841. (3). The Caris POA Retrospective Repository (COE-002-0116) was approved by WIRB (WIRB Tracking #20162657) and has an NCT# of NCT 00326499. (4). ION data is covered under an IRB exemption. All data are retrospective and have been de-identified prior to Caris receiving it and authors performing independent analyses.

**CONFLICTS OF INTEREST**

No authors declare a conflict of interest and received no honoraria for these publications.

Original article: Oncotarget. 2018; 9:11371-11376. https://doi.org/10.18632/oncotarget.24257

